# Reply to Comments on the editor Re: Carsten Posovszky et al. “Roles of Lactose and Fructose Malabsorption and Dietary Outcomes in Children Presenting with Chronic Abdominal Pain.”, *Nutrients* 2019, *11*(12), 3063

**DOI:** 10.3390/nu12061556

**Published:** 2020-05-27

**Authors:** Carsten Posovszky, Vreni Roesler, Sebastian Becker, Enno Iven, Christian Hudert, Friedrich Ebinger, Claudia Calvano, Petra Warschburger

**Affiliations:** 1 University Medical Centre Ulm, Department for Pediatric and Adolescent Medicine, Pediatric Gastroenterology, 89075 Ulm, Germany; vreni.roesler@uniklinik-ulm.de; 2 Darmstädter Kinderkliniken Prinzessin Margaret, Dieburger Str. 31, 64287 Darmstadt, Germany; Sebastian.Becker@alice-hospital.de; 3 Katholisches Kinderkrankenhaus Wilhelmsstift, Liliencronstr.130, 22149 Hamburg, Germany; e.iven@kkh-wilhelmstift.de; 4 Charité–Universitätsmedizin Berlin, corporate member of Freie Universität Berlin, Humboldt-Universität zu Berlin and Berlin Institute of Health, Department of Pediatric Gastroenterology, Augustenburger Platz 1, 13353 Berlin, Germany; christian.hudert@charite.de; 5 St. Vincenz-Krankenhaus GmbH Paderborn, Klinik für Kinder- und Jugendmedizin, Husener Str. 81, 33098 Paderborn, Germany; F.Ebinger@vincenz.de; 6 Charitü–Universitätsmedizin Berlin, corporate member of Freie Universität Berlin, Humboldt-Universität zu Berlin and Berlin Institute of Health, Department of Child and Adolescent Psychiatry, Augustenburger Platz 1, 13353 Berlin, Germany; claudia.calvano@charite.de; 7 Department Psychology, University of Potsdam, Counseling Psychology, Karl-Liebknecht-Str. 24-25, 14476 Potsdam, Germany; warschb@uni-potsdam.de

We would like to thank Drs. Hammer and Hammer for their interest in our work and their comments [1] on our recent paper [2]. The following is our response to their concerns.

First, the authors commented on the distinction between malabsorption and intolerance used in our study. Food intolerance is defined as a non-immunological response initiated by a food product or component at a dose normally tolerated and accounts for most adverse food responses [3]. Carbohydrate malabsorption is the physiologic problem after ingestion that can manifest as intolerance characterized by a clinical syndrome of at least one of the following: abdominal pain, diarrhea, nausea, flatulence and/or bloating [4,5,6,7,8]. Accordingly, the diagnosis of carbohydrate intolerance in our study was based on a positive hydrogen breath test (H_2_BT) with typical symptoms after fructose and/or lactose load and confirmed by response to an open label elimination diet at follow-up. Furthermore, the attending physician determined the leading cause of chronic abdominal pain (CAP) by evaluating the reported frequency and intensity of abdominal pain and other GI symptoms in relation to dietary intake of fructose or lactose. This trial-and-error approach is widely accepted to confirm food intolerance [3]. Accordingly, carbohydrate intolerance was initially diagnosed in 55 out of 135 children after fructose and/or lactose load. However, relief of abdominal pain after open-label elimination of the respective carbohydrates was only reported in 10 of these children in the follow-up of our study. This observation indicates that we may erroneously diagnose carbohydrate intolerance by performing breath tests with unusually high exposure doses e.g., in children with a predominant functional abdominal pain disorder (FADP). Moreover, in a well-designed placebo-controlled trial neither lactose nor fructose intolerance were finally proven as cause of recurrent abdominal pain after H_2_BT, open elimination diet and provocation, and double-blinded placebo-controlled provocation [9]. Therefore, we need to discriminate between carbohydrate intolerance as an exclusive cause of chronic abdominal pain (CAP) or detection of breath test related symptomatic malabsorption in predominant FAPD. Gijsbers et al. similarly discriminated between carbohydrate malabsorption and intolerance in their study [9]. Second, the authors questioned the validity of symptom assessment during breath tests as well as after elimination diet. We carefully assessed symptoms throughout H_2_BT by means of a standardized documentation sheet and by face-to-face interview after elimination diet as recommended and performed in many other studies [10,11]. We agree with the authors that a validated instrument for unbiased symptom assessment is essential and appreciate their efforts in establishing and validating a standardized questionnaire for symptom assessment before and after carbohydrate breath test [12]. In addition, Glatstein assessed symptoms before and after hydrogen breath test using a questionnaire and a validated pain scale [13]. These instruments were not available when we performed our study. Furthermore, to our knowledge both questionnaires have not been validated by other groups yet. 

Third, the authors were concerned whether the patients with a negative breath test did develop abdominal symptoms after the test. Symptomatic patients without relevant increases in exhalated hydrogen have been frequently described [9,12]. Indeed, we also observed abdominal symptoms in patients with negative breath tests but this was not mentioned in our publication. However, an interpretation of abdominal symptoms in children with CAP not displaying a significant increase of hydrogen may lead to wrong assumptions. For example, abdominal pain was reported as the least specific symptom of lactose intolerance [13]. 

Fourth, the authors questioned the definition of carbohydrate intolerance as an organic disease instead of a functional disorder. We referred to a well-established definition of functional abdominal pain based on Rome III when developing the protocol for this trial [14,15]. Indeed, in Rome IV the phrase “no evidence of an inflammatory, anatomic, metabolic, or neoplastic process that explain the subject’s symptoms” has been removed from the diagnostic criteria for functional disorders and changed to Rome III’s denomination of abdominal pain related functional disorders to functional abdominal pain disorder (FAPD) [16]. Thus, the dictum that there is no evidence of organic disease has been replaced by the demand that “after appropriate medical evaluation the symptoms cannot be attributed to another medical condition” [16]. We carefully discriminated between patients with carbohydrate intolerance as the exclusive factor for their abdominal pain distress, which resolves completely after appropriate diet, and patients with malabsorption but other medical conditions (e.g., celiac disease) responsible for their suffering and the coexistence of lactose/fructose malabsorption and FAPD. This classification is of clinical impact in order to select children with predominant FAPD for a cognitive behavioral intervention [17]. There is an erratum in Table 3 in the right column of the category malabsorption: FAPD must appear instead of OAPD.

In conclusion, we address the necessity of a differentiated approach in diagnosis of CAP in children to find a reliable diagnosis and the appropriate therapy more efficiently and to avoid unnecessary burden on the patients. Our study highlights the potential contribution of fructose and lactose intolerance to CAP in children and critically reflects on the reliability of hydrogen breath testing in this context. We agree that validated symptom and dietary assessment is essential in order to confirm the diagnosis of carbohydrate intolerance especially during follow-up. We acknowledge that a strict separation of carbohydrate intolerance into functional and organic disorders on a biological basis is not feasible. 

Given the lack of understanding both in terms of the mechanisms involved in the generation of symptoms resulting from carbohydrate malabsorption and the effect of dietary restrictions (e.g., FODMAP diet) in children with FAPD stress, further studies in that field are warranted.
